# Microbiological Surveillance of Peritoneal Dialysis Associated Peritonitis: Antimicrobial Susceptibility Profiles of a Referral Center in GERMANY over 32 Years

**DOI:** 10.1371/journal.pone.0135969

**Published:** 2015-09-25

**Authors:** Daniel Kitterer, Joerg Latus, Christoph Pöhlmann, M. Dominik Alscher, Martin Kimmel

**Affiliations:** 1 Department of Internal Medicine, Division of Nephrology, Robert-Bosch-Hospital, Stuttgart, Germany; 2 Department of Diagnostic and Laboratory Medicine, Robert-Bosch-Hospital, Stuttgart, Germany; University Hospital of the Albert-Ludwigs-University Freiburg, GERMANY

## Abstract

**Objectives:**

Peritonitis is one of the most important causes of treatment failure in peritoneal dialysis (PD) patients. This study describes changes in characteristics of causative organisms in PD-related peritonitis and antimicrobial susceptibility.

**Methods:**

In this single center study we analyzed retrospective 487 susceptibility profiles of the peritoneal fluid cultures of 351 adult patients with peritonitis from 1979 to 2014 (divided into three time periods, P1-P3).

**Results:**

*Staphylococcus aureus* decreased from P1 compared to P2 and P3 (P<0.05 and P<0.01, respectively). Methicillin-resistant *S*. *aureus* (MRSA) occurred only in P3. Methicillin-resistant *Staphylococcus epidermidis* (MRSE) increased in P3 over P1 and P2 (P <0.0001, respectively). In P2 and P3, vancomycin resistant enterococci were detected. The percentage of gram-negative organisms remained unchanged. Third generation cephalosporin resistant gram-negative rods (3GCR-GN) were found exclusively in P3. Cefazolin-susceptible gram-positive organisms decreased over the three decades (93% in P1, 75% in P2 and 58% in P3, P<0.01, P<0.05 and P<0.0001, respectively). Vancomycin susceptibility decreased and gentamicin susceptibility in gram-negatives was 94% in P1, 82% in P2 and 90% in P3. Ceftazidim susceptibility was 84% in P2 and 93% in P3.

**Conclusions:**

Peritonitis caused by MSSA decreased, but peritonitis caused by MRSE increased. MRSA peritonitis is still rare. Peritonitis caused by 3GCR-GN is increasing. An initial antibiotic treatment protocol should be adopted for PD patients to provide continuous surveillance.

## Introduction

Since continuous ambulatory peritoneal dialysis (CAPD) was invented in the early 80`s of the last century, peritoneal dialysis (PD) has become a standard of care in end-stage renal disease (ESRD) patients [1]. Approximately 200,000 patients worldwide are treated with PD, which represents 11% of the global dialysis population [2]. However, peritonitis remains one of the principal complication, despite all reasonable preventative strategies [3]. The incidence of PD-associated peritonitis varies from 0.06–1.66 episodes/patient-year in different centers and different countries [4]. Peritonitis can lead to treatment failure and termination of the procedure in 20% of patients and has a mortality of 2–6% [5, 6]. Empirical antibiotic strategies must cover both gram-positive and gram-negative organisms. The Society for Peritoneal Dialysis (ISPD) has recommended center-specific empirical therapy. The antibiotic regimen should be selected according to local antimicrobial susceptibility profiles [7]. Intraperitoneal administration of antibiotics was superior to intravenous administration for treating PD-associated peritonitis [8, 9]. The ISPD guidelines emphasize the need to monitor resistance among various gram-negative bacteria [4, 7]. It is therefore essential to develop and implement an antibiotic stewardship program to improve empirical therapy and to monitor the resistance rates in PD-associated peritonitis.

Here we report the antimicrobial susceptibility profiles and distribution of causative organisms in our referral center in Germany over more than three decades.

## Materials and Methods

### Ethical Considerations

The Ethical Commission of the Medical University of Tuebingen approved this study (Ethical Approval/Registration Number: 103/2015R). The analysis was performed retrospective without written or verbal informed consent to participate in this study. Due to the long study period and the retrospective anonymous analysis of the data, nevertheless the lack of informed consent has been approved by the local ethics committee.

### Study Population

We analyzed all peritonitis episodes from January 1979 to December 2014 at the Robert-Bosch-Hospital, Stuttgart, Germany. Peritonitis was defined as the presence of abdominal pain and/or cloudy effluent with a cell count greater than 100/μL, with at least 50% polymorphonuclear cells as stated in the current guidelines of the International Society for Peritoneal Dialysis (ISPD) [7]. A systematic review of all peritoneal fluid cultures (PFC) was performed in our laboratory. During this period, 487 PFC from 351 adult patients were documented, of which 61 PFC were culture negative (12.5%) and were excluded from the study (P1 = 12 culture negative episodes, ratio culture positive/culture negative 10:1; P2 = 15 culture negative episodes, ratio culture positive/culture negative 8:1; P3 = 34 culture negative episodes, ratio culture positive/culture negative 7:1). Episodes with peritoneal eosinophilia and negative PFC were also excluded from the study. Nasal mupirocin application in all *S*. *aureus* carriers began in 1994. Since the early 90s, PD nurses provide intensive training in every „new”PD patient in our center. In patients with *S*. *aureus* exit-site colonization topical antibiotic at the catheter exit in agreement with the current ISPD guidelines were used [7]. Prior to 1994, no antibiotic therapy was used as a prophylaxis for peritonitis.

#### Microbiological specimen collection, antimicrobial susceptibility testing and data analyses

The abdomen was drained and effluent was collected from the patient’s outlet bag. Dialysate effluent was collected under sterile conditions and 10ml effluent was incubated per blood culture bottle (BCB) for at least two BCBs (aerobic/anaerobic) as recommended by the ISPD Guidelines [7, 10, 11]. BCBs were used from bioMérieux (Germany) and BD (Germany) and were routinely incubated for seven days. Positive BCBs were subcultured on both aerobic and anaerobic standard media. Etiologic identification of grown organisms was performed employing specific metabolic reactions in conventional manual (Api test systems, bioMérieux, Germany) or automated test systems (VITEK 2, bioMérieux, Germany). Since 2011, identification of bacteria was primarily done using the MALDI Biotyper System (Bruker Corporation Billerica, *USA)*. The VITEK 2 system, which was implemented in 2003, was also used for susceptibility testing of bacteria. Before 2003, agar diffusion was the dominant technique for sensitivity testing. However, until now it is still used in the minority of cases due to its simplicity. If the agar diffusion technique was used, no minimum inhibitory concentration (MIC) was available. *In Vitro* susceptibility was defined according to the limit specifications (breakpoints) of the Clinical and Laboratory Standards Institute (CLSI) [12]. Strains presenting intermediate values were considered to be resistant. For comparison, the PFC samples were divided into three periods (2from 1979–1991, period 2 1993–2003, period 3 2004–2014). The susceptibility profiles of each organism were determined from the data.

#### Empiric therapy during the study period

From 1979 to 2011 the empiric therapy at our center was cefazolin intraperitoneal (i.p.) and gentamycin i.p. in PD patients with peritonitis. After interim analysis of the shown microbiological data, empiric therapy was changed to vancomycin i.p. and gentamycin i.p. in 2014.

### Statistical Analyses

Comparisons between the three groups were performed with the Fisher’s exact test (two-tailed). Analysis was performed using the statistical software package Prism (GraphPad San Diego, California, USA). *P* < 0.05 was considered significant.

## Results

### Distribution of Organisms

**Fig 1:**
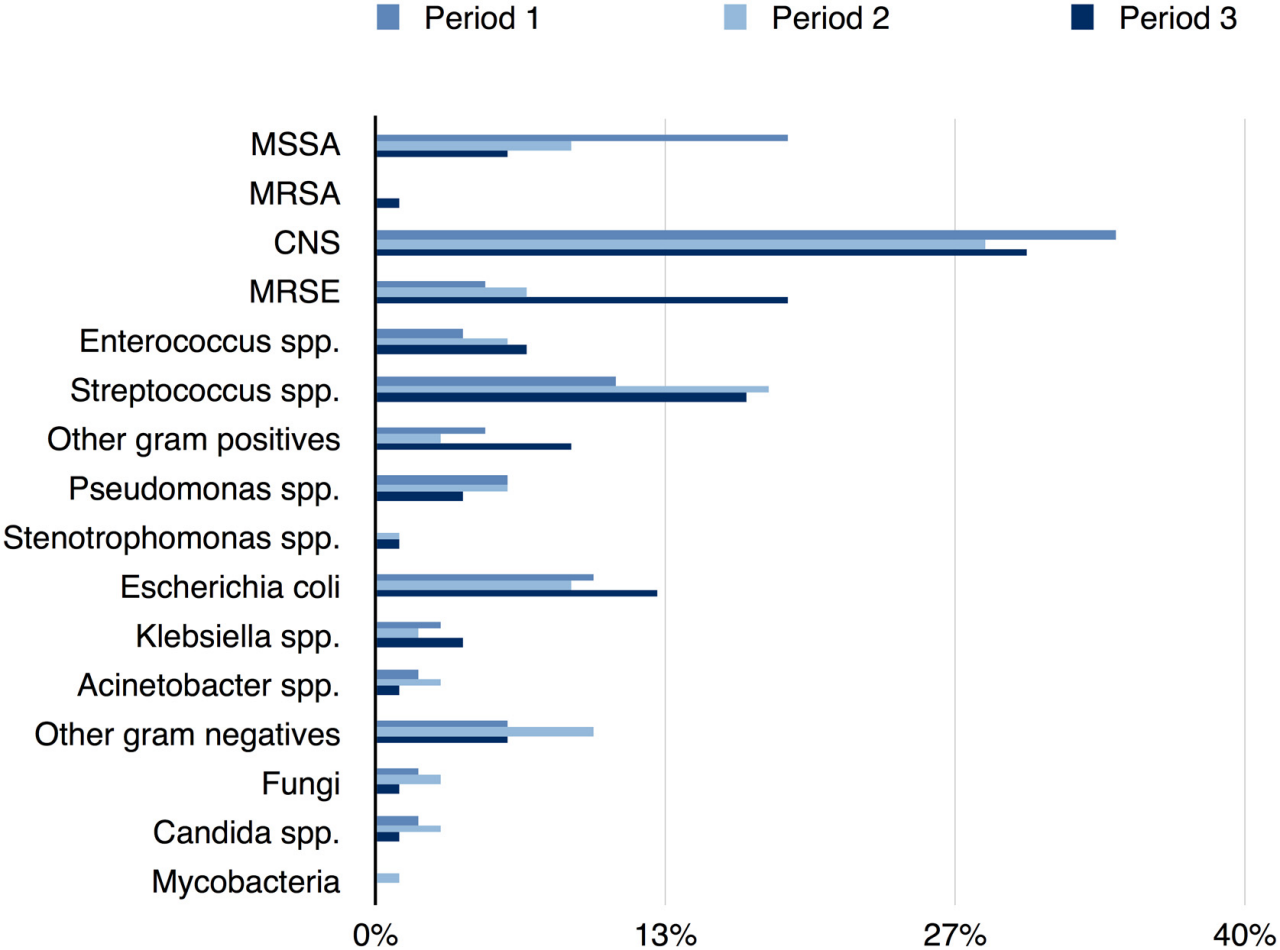
Etiologic Spectrum of three different Peritonitis Episodes over 32 years. Distribution of organisms in period 1 (1979–1992), period 2 (1993–2003) and period 3 (2004–2014); all variables are expressed as percentage; Abbreviation: MSSA, methicillin-sensitive *Staphylococcus aureus*; MRSA, methicillin-resistant *Staphylococcus aureus*; CNS, coagulase-negative staphylococci; MRSE, methicillin-resistant *Staphylococcus epidermidis*.

We analyzed 487 susceptibility profiles of patients with PD-associated peritonitis since the PD was begun in January 1979 at the Robert-Bosch-Hospital, Germany. In P1, 66 PD patients were treated (in total 120 peritonitis episodes) in our center. In P2, the number of PD patients remained stable (69 patients with 125 peritonitis episodes). The number of PD patients increased in P3 (130 PD patients with 241 peritonitis episodes). Over the whole study period (P1-P3) there was a slight increase of gram-negative bacteria and a slight decrease of gram-positive bacteria. The ratio of gram-negative to gram-positive bacteria increased from 0.4 in P1 to 0.5 in P3 (p > 0.05, Fig 1).

#### Distribution of Gram-positive organisms

Gram-positive organisms caused 70% of the peritonitis episodes. In period 1, we found a significant surplus of *Staphylococcus aureus* compared to period 2 and period 3 as shown in Table 1 (P<0.05 and P<0.01, respectively). In period 1 and period 2 only methicillin-sensitive *S*. *aureus* (MSSA) strains could be detected. In the last period, two strains of methicillin-resistant *S*. *aureus* were found (1% of all episodes). Simultaneously, the proportion of methicillin-resistant *Staphylococcus epidermidis* (MRSE) increased in period 3 compared to period 1 and 2 (P<0.0001, respectively). The percentage of *Enterococcus* spp. increased from 4% to 7% over the three decades, but the difference was not statistically significant(period 1 compared to period 3). In period 1, no vancomycin resistant enterococcus (VRE) was detected. However in period 2 and period 3, VRE appeared (13% and 19% of all *Enterococcus* spp., respectively), but the difference was not statistically significant. The percentage of gram-negative organisms remained unchanged over the three decades (Table 1, Fig 1).

**Table 1:** Etiologic Spectrum of three different Peritonitis Episodes over 32 years.

Organisms identified	Number of episodes		
	Period 1 [n (%)]	Period 2 [n (%)]	Period 3 [n (%)]
**Gram-positive organisms**	87 (73)	81 (65)	168 (69)
*Staphylococcus aureus*	23 (19)^1^	11 (9)	17 (7)
MSSA	23 (19)	11 (9)	15 (6)
MRSA	0 (0)	0 (0)	2 (1)
*CNS*	41 (34)	35 (28)	73 (30)
MRSE	6 (5)	9 (7)	45 (19)^2^
*Enterococcus* spp.	5 (4)^a^ ^,^	8 (6)^b^ ^,^	16 (7)^c^ ^,^
*Streptococcus* spp.	13 (11)	23 (18)	40 (17)
Other gram positive rods	2 (2)	3 (2)	17 (7)
Others	3 (3)	1 (1)	5 (2)
**Gram-negative organisms**	31 (26)	39 (31)	71 (29)
*Pseudomonas* spp.	7 (6)	8 (6)	10 (4)
*Stenotrophomonas* spp.	0 (0)	1 (1)	3 (1)
*Escherichia coli*	12 (10)^d^	11 (9)^d^	31 (13)^e^
*Klebsiella* spp.	3 (3)^d^	2 (2)^d^	9 (4)^f^
*Acinetobacter* spp.	2 (2)^d^	4 (3)^d^	3 (1)^g^
Others	7 (6)^d^	13 (10)^d^	15 (6)^h^
**Fungi**	2 (2)	4 (3)	3 (1)
*Candida* spp.	2 (2)	4 (3)	3 (1)
**Mycobacteria**	0 (0)	1 (1)	0 (0)
Total Episodes	120	125	242

MSSA, methicillin-sensitive *S*. *aureus*; MRSA, methicillin-resistant *S*. *aureus*; CNS, coagulase-negative staphylococci; MRSE, methicillin-resistant *Staphylococcus epidermidis*; Period 1 (1979–1992); Period 2 (1993–2003); Period 3 (2004–2014);

^a^no vancomycin resistant enterococcus (VRE);

^b^13% VRE;

^c^19% VRE;

^d^ third generation cephalosporin resistant gram-negative rods (3GCR-GN);

^e^6.5% 3GCR-GN;

^f^22.2% 3GCR-GN;

^g^ 33.3% 3GCR-GN;

^h^ 6.7% 3GCR-GN;

^1^period 1 vs period 2 and period 1 vs period 3 P<0.05;

^2^ period 3 vs period 1 and 2 p <0.0001; variables are expressed in number of episodes (percentage in parentheses);

#### Distribution of SPICE (*Serratia*, *Pseudomonas/Providencia*, indole-positive *Proteus/ Acinetobacter/Morganella*, *Citrobacter*, *Enterobacter* or *Hafnia*) and third generation cephalosporin resistant organisms

Period 1: Two *Acinetobacter* spp. (2%), one *Serratia* spp., one *Citrobacter* spp. (0.8%, respectively) and three *Enterobacter* spp. (2.5%) occurred.

Period 2: Four *Acinetobacter* spp. (3%), one *Proteus* spp. (0.8%), one *Serratia* spp. (0.8%, four *Citrobacter* spp. (3.2%), one *Enterobacter* spp. (0.8%) and one *Morganella* spp. (0.8%) occurred.

*Period 3*: Three *Acinetobacter* spp. (1%), four *Serratia* spp. (1.7%), two *Citrobacter* spp. (0.8%) and three *Enterobacter* spp. (1.2%) occurred. Furthermore, two *E*. *coli* (6.5%), two *Klebsiella* spp. (22.2%), one *Acinetobacter baumannii* (33.3%) and one *Aeromonas hydrophila* (6.7% of other gram-negative organisms) were classified as being resistant against third generation cephalosporin (3GCR-GN), mainly due to ESBL and/or derepressed AmpC phenotypes. All strains were susceptible to imipenem. Proportions of the various microorganisms are shown in Table 1.

### *In Vitro* Susceptibility Rates

The *in vitro* susceptibilities observed in the three periods are shown in Tables 2 and 3, respectively. For MSSA, the proportion of bacteria susceptible to gentamicin was statistically different between period 1 and period 2 (Table 2). Susceptibility to gentamicin was 70% in P1 and increased to 100% in P2. For doxycycline, the proportion of susceptible strains increased from 70% in period 1 to 100% in period 3. The difference was statistically significant (P<0.05). In *Streptococcus* spp. susceptibility to Levofloxacin increased from 33% in period 1 to 82% in P3 (P<0.001). In *E*. *coli*, susceptibility to trimethoprim/sulfamethoxazole (TXM-SMX) decreased from 83% in period 1 to 63% period 2. In period 3, susceptibility to TXM-SMX increased to 84% (P<0.0001).

**Table 2:** Gram-positive organisms causing Peritoneal Dialysis-Related Peritonitis and their *In Vitro* Susceptibility Rates over 32 years.

	Methicillin-sensitive *S*. *aureus* n (% susceptible)				Methicillin-resistant *S*. *epidermidis* n (% susceptible)				*Enterococcus* spp. n (% susceptible)				*Streptococcus* spp. n (% susceptible)			
	P1	P2	P3	*P*	P1	P2	P3	*P*	P1	P2	P3	*P*	P1	P2	P3	*P*
Ampicillin	23 (52)	11 (36)	15 (47)	NS	6 (0)	9 (0)	45 (0)	NS	5 (100)	8 (63)	16 (69)	NS	13 (92)	22 (95)	40 (93)	NS
Amp/Sulb	NT	10 (90)	15 (100)	---	NT	9 (0)	45 (0)	NS	NT	NT	NT	---	NT	22 (100)	36 (100)	NS
Cefazolin	23 (100)	11 (91)	15 (100)	NS	6 (0)	9 (0)	45 (0)	---	NT	NT	NT	---	13 (92)	NT	NT	---
Ceftriaxon	NT	2 (50)	NT	---	NT	1 (0)	NT	---	NT	NT	NT	---	NT	8 (100)	39 (100)	NS
Vancomycin	17 (100)	11 (100)	15 (100)	NS	6 (100)	9 (100)	45 (100)	NS	NT	8 (88)	16 (81)	NS	13 (100)	23 (100)	39 (100)	NS
Linezolid	NT	3 (100)	14 (100)	---	NT	3 (100)	45 (100)	NS	NT	5 (100)	16 (100)	NS	NT	1 (100)	3 (100)	---
Imipinem	14 (100)	11 (91)	15 (100)	NS	NT	9 (0)	45 (0)	NS	NT	8 (63)	15 (73)	NS	5 (100)	22 (100)	40 (100)	NS
Gentamicin	23 (70)	11 (91)	15 (100)	<0.05*	6 (67)	9 (67)	45 (56)	NS	5 (80)	8 (63)	15 (53)	NS	13 (54)	16 (25)	9 (44)	NS
Moxifloxacin	NT	NT	14 (100)	---	NT	NT	45 (13)	---	NT	NT	15 (40)	---	NT	NT	35 (89)	---
Levofloxacin	NT	8 (100)	15 (100)	NS	NT	8 (13)	42 (12)	NS	NT	6 (83)	15 (40)	NS	NT	15 (33)	34 (82)	<0.001
Doxycycline	23 (70)	11 (91)	15 (100)	<0.05*	6 (100)	9 (78)	45 (84)	NS	5 (40)	5 (100)	12 (58)	NS	13 (85)	21 (48)	35 (63)	NS
*Episodes*	*23*	*11*	*15*		*6*	*9*	*45*		*5*	*8*	*16*		*13*	*23*	*40*	

P1 = (1979–1992); P2 = period 2 (1993–2003); P3 = period 3 (2004–2014); number of antibiograms; percentage of full susceptibility; NT, not tested; Amp/Sulb, ampicillin/sulbactam,

*comparison between period 1 and period 3; NS, not significant;

**Table 3:** Gram-negative organisms causing Peritoneal Dialysis-Related Peritonitis and their *In Vitro* Susceptibility Rates over 32 years.

	*Pseudomonas* spp. n (% susceptible)				*Klebsiella* spp. n (% susceptible)				*Escherichia coli* n (% susceptible)				*Other Gram-neg*. *organisms* ^*##*^. n (% susceptible)			
	P1	P2	P3	*P*	P1	P2	P3	*P*	P1	P2	P3	P	P1	P2	P3	*P*
Ampicillin	7 (57)	NT	1 (100)^a^	---	3 (0)	2 (0)	9 (0)	---	12 (67)	11 (45)	28 (46)	NS	7 (14)	12 (8)	11 (27)	NS
Amp/Sulb	NT	NT	1 (100)^a^	---	NT	2 (50)	9 (78)	---	NT	9 (67)	30 (80)	---	NT	11 (9)	11 (57)	NS
Cefotaxim	7 (57)	1 (0)	NT	---	3 (100)	1 (100)	9 (78)	NS	12 (100)	2 (100)	27 (89)	NS	5 (100)	NT	11 (91)	NS
Ceftazidime	2 (100)	8 (75)	10 (100)	NS	NT	2 (100)	9 (78)	NS	1 (100)	11 (100)	31 (94)	NS	NT	12 (100)	14 (93)^b^	NS
Cefepime	NT	2 (50)	10 (100)	---	NT	1 (100)	8 (75)	---	NT	2 (100)	31 (97)	---	NT	NT	14 (93)^b^	---
Gentamicin	7 (86)	8 (100)	10 (90)	NS	3 (100)	2 (50)	9 (89)	NS	12 (100)	11 (82)	31 (97)	NS	7 (100)	12 (83)	14 (71)^b^	NS
Imipinem	4 (100)	8 (100)	10 (100)	NS	2 (100)	2 (100)	9 (100)	NS	11 (100)	11 (100)	31 (100)	NS	1 (100)	12 (92)	14 (100)^b^	NA
Ofloxacin	NT	NT	NT		NT	1 (100)	NT	---	NT	2 (100)	NT	---	NT	7 (100)	NT	---
Ciprofloxacin	NT	8 (100)	10 (80)	NS	NT	1 (100)	9 (89)	---	1 (100)	4 (75)	31 (87)	---	NT	NT	14 (57)	---
Levofloxacin	NT	8 (100)	10 (80)	NS	NT	1(100)	9 (89)	---	NT	8 (63)	31 (84)	NS	NT	5 (100)	14 (57)^b^	NS
TMX-SMX	7 (57)	1 (0)	3 (33)	NS	3 (100)	2 (100)	9 (100)	NS	12 (83)	11 (64)	31 (84)	<0.0001^#^	6 (83)	12 (67)	13 (77)	NS
*Episodes*	*7*	*8*	*10*		*3*	*2*	*9*		*12*	*11*	*31*		*7*	*13*	*15*	

P1 = period 1 (1979–1992); P2 = period 2 (1993–2003); P3 = period 3 (2004–2014); NT, not tested; number of antibiograms; percentage of full susceptibility; Amp/Sulb, ampicillin/sulbactam; TMX-SMX, trimethoprim/sulfamethoxazole; NA, not available;

^##^including Enterobacteriaceae (except Eschericha coli and Klebsiella supp.), except Acinetobacter spp.;

^a^Pseudomonas oryzihabitans;

^b^Neisseria spp. was not tested;

^#^comparison between period 2 and period 3; NS, not significant;

### *In Vitro* Susceptibility Rates to empiric initial intraperitoneal therapies.

#### Gram-positive organisms

**Fig 2:**
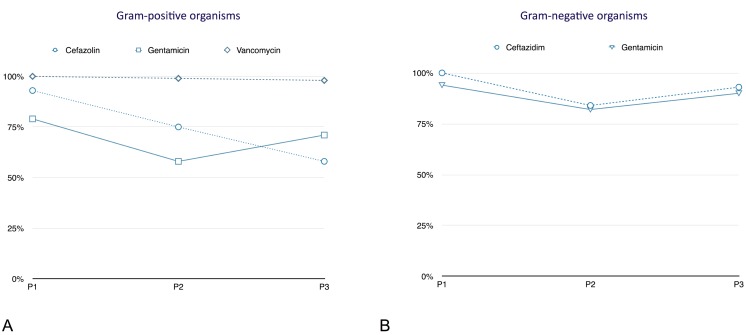
In vitro susceptibility to empirical intraperitoneal treatment. A. Gram-positive organisms; B. Gram-negative organisms; P1, period 1 (1979–1992), n = 120 episodes; P2, period 2 (1993–2003), n = 125 episodes; P3, period 3 (2004–2014), n = 242 episodes; all variables are expressed as percentage.

In period 1, the susceptibility rate to cefazolin was 93%. In period 2, the susceptibility rate decreased to 75% and in P3 to 58% (P<0.01, P<0.05 and P<0.0001, respectively). In period 1, 79% of all gram-positive strains were sensitive to gentamicin. In period 2, the susceptibility decreased to 58% and in period 3 the susceptibility increased to 71%. The difference between period 1 and period 3 was not significant (P = 0.2). Sensitivity to vancomycin was almost equal over the three periods (98–100%) (Tables 4–6, Fig 2).

**Table 4:** *In Vitro* Susceptibility Rates of different pathogens to empiric initial intraperitoneal therapies in period 1 (1979–1992).

1979–1992	Cefazolin n (% susceptible)	Ceftazidim n (% susceptible)	Gentamicin n (% susceptible)	Vancomycin n (% susceptible)
**Gram-positive organisms**	82 (93)	7 (86%)	82 (79)	80 (100)
*Staphylococcus aureus*	23 (100)	4 (100)	23 (70)	17 (100)
MSSA	23 (100)	4 (100)	23 (70)	17 (100)
MRSA	NA	NA	NA	NA
*CNS*	41 (95)	NT	41 (68)	16 (100)
MRSE	NT	NT	6 (67)	6 (100)
*Enterococcus* spp.	NT	NT	5 (80)	NT
*Streptococcus* spp.	13 (85)	3 (67)	13 (54)	6 (100)
Other gram positive rods	2 (100)	NT	2 (100)	2 (100)
Others	3 (67)	NT	3 (100)	1 (100)
**Gram-negative organisms**	NT	3 (100)	32 (94)	NT
*Pseudomonas* spp.	NT	3 (100)	7 (86)	NT
*Stenotrophomonas* spp.	NT	N	NA	NT
*Escherichia coli*	NT	NT	12 (100)	NT
*Klebsiella* spp.	NT	NT	3 (100)	NT
*Acinetobacter* spp.	NT	NT	3 (100)	NT
Others	NT	NT	7 (86)	NT

MSSA, methicillin-sensitive *S*. *aureus*; MRSA, methicillin-resistant *S*. *aureus*; CNS, coagulase-negative staphylococci; NA, not available, NT, not tested; variables are expressed in number of antibiograms (percentage in parentheses regarding Susceptibility Rate);

**Table 5:** *In Vitro* Susceptibility Rates of different pathogens to empiric initial intraperitoneal therapies in period 2 (1993–2003).

1993–2003	Cefazolin n (% susceptible)	Ceftazidim n (% susceptible)	Gentamicin n (% susceptible)	Vancomycin n (% susceptible)
**Gram-positive organisms**	81 (75)	4 (25)	73 (58)	81 (99)^a^
*Staphylococcus aureus*	11 (91)	NT	11 (91)	11 (100)
MSSA	11 (91)	NT	11 (91)	11 (100)
MRSA	NA	NA	NA	NA
*CNS*	35 (63)	NT	35 (60)	35 (100)
MRSE	NT	NT	9 (67)	9 (100)
*Enterococcus* spp.	NT	NT	8 (63)	8 (88)^a^
*Streptococcus* spp.	22 (91)	NT	15 (20)	23 (100)
Other gram positive rods	3 (67)	2 (0)	3 (67)	3 (100)
Others	1 (0)	NT	1 (0)	1(100)
**Gram-negative organisms**	NT	38 (84)	38 (82)	NT
*Pseudomonas* spp.	NT	8 (75)	8 (100)	NT
*Stenotrophomonas* spp.	NT	1 (0)	1 (0)	NT
*Escherichia coli*	NT	11 (100)	11 (82)	NT
*Klebsiella* spp.	NT	2 (100)	2 (50)	NT
*Acinetobacter* spp.	NT	4 (25)	3 (75)	NT
Others	NT	12 (100)^b^	12 (83)^b^	NT

MSSA, methicillin-sensitive *S*. *aureus*; MRSA, methicillin-resistant *S*. *aureus*; CNS, coagulase-negative staphylococci; MRSE, methicillin-resistant Staphylococcus epidermidis; NA, not available; NT, not tested;

^a^ one vancomycin resistant enterococcus (VRE);

^b^ Bacteroides spp.; variables are expressed in number of antibiograms (percentage in parentheses regarding Susceptibility Rate);

**Table 6:** *In Vitro* Susceptibility Rates of different pathogens to empiric initial intraperitoneal therapies in period 3 (2004–2014).

2004–2014	Cefazolinn n (% susceptible)	Ceftazidimn n (% susceptible)	Gentamicin n (% susceptible)	Vancomycin n (% susceptible)
**Gram-positive organisms**	113 (58)	5 (100)^a^	130 (71)	156 (98)^b^
*Staphylococcus aureus*	17 (88)	NT	17 (88)	17 (100)
MSSA	15 (100)	NT	15 (94)	15 (100)
MRSA	2 (0)	NT	2 (50)	2 (100)
*CNS*	73 (38)	NT	73 (70)	70 (100)
MRSE	NT	NT	45 (56)	45 (100)
*Enterococcus* spp.	NT	NT	16 (53)	16 (81)^b^
*Streptococcus* spp.	19 (95)	NT	10 (40)	39 (100)
Other gram positive rods	2 (0)	2 (100)^a^	10 (70)	10 (100)
Others	1 (100)	3 (100)^a^	3 (100)	4 (100)
**Gram-negative organisms**	NT	68 (93)	68 (90)	NT
*Pseudomonas* spp.	NT	10 (100)	10 (90)	NT
*Stenotrophomonas* spp.	NT	1 (100)	1 (100)	NT
*Escherichia coli*	NT	31 (94)	31 (94)^c^	NT
*Klebsiella* spp.	NT	9 (78)^c^	9 (89)	NT
*Acinetobacter* spp.	NT	3 (33)	3 (100)	NT
Others	NT	14 (93)	14 (71)	NT

MSSA, methicillin-sensitive *S*. *aureus;* MRSA, methicillin-resistant *S*. *aureus*; CNS, coagulase-negative staphylococci; MRSE, methicillin-resistant Staphylococcus epidermidis; NA, not available; NT, not tested;

^a^ *Gemella morbillorum*, *Micrococcus luteus*

^b^Vancomycin resistant enterococcius (VRE);

^c^third generation cephalosporin resistant gram-negative rods (3GCR-GN); variables are expressed in number of antibiograms (percentage in parentheses regarding Susceptibility Rate);

#### Gram-negative organisms

The response to ceftazidim was 100% in period 1 (three patients). In period 2, susceptibility to ceftazidim was 84% and in period 3 it was 93%. The difference was not statistically significant (P = 0.2). Gram-negative strains were sensitive to gentamicine in 94% of episodes in period 1. In period 2, susceptibility to gentamicine decreased to 82% and in period 3, 90% of all gram-negative organisms were susceptible to gentamicine (all P> 0.05) (Tables 4–6, Fig 2).

## Discussion

In this study we focused on the microbiological pattern with corresponding *in vitro* antibiotic susceptibility rates for PD-associated peritonitis over three decades in our center in Germany. To our knowledge, this analysis of the distribution of causative organisms in PD-related peritonitis and antimicrobial susceptibility profiles covers the longest time period studied. Over the study period we found a slight, non-significant, increase of gram-negative bacteria and a slight decrease of gram-positive bacteria reflected by an increase of the ratio from gram-negative to gram-positive bacteria (0.4 in P1 to 0.5 in P3).

In our study population, *S*. *aureus* decreased from period 1 to period 3. Scottish data reported a decrease in *S*. *aureus*-associated peritonitis over a four-year period [13], whereas a study from Korea reported no decrease over a decade [14]. It was postulated that the introduction of the double-bag (twin-bag) system in the 1980s, technical evolutions, *S*. *aureus* screening and treatment with mupirocin was responsible for this development [15–17]. Proportions of peritonitis-associated CNS episodes declined from 29% in period 1 to 12% in period 3 in our patients. This agrees well with previous studies [18]. On the other hand, the proportions of MRSE episodes increased over the three decades to 19% as described for other populations [14, 19]. Interestingly, MRSA associated peritonitis remains a rare event with only two episodes in period 3. In our study, susceptibility to cefazolin in gram-positive organisms decreased over the three decades (period 1 compared to period 2), but the difference was not statistically significant. On the other hand the percentage of *Enterococcus* spp. varied between 4% and 7%. This indicates a favor for treatment with vancomycin as a first line antibiotic regimen in our in hospital patient cohort. Though in period 2 and period 3 VRE appeared, this is an important point that must be considered if initial treatment is not effective. Gram-negative organisms are often resistant to antibiotics because of either an plasmid encoded beta lactamase (e.g., extended beta lactamase (ESBL) producers) or chromosomally mediated beta-lactamases (e.g. derepressed AmpC beta-lactamase); these organisms are summarized by the acronym SPICE (*Serratia*, *Pseudomonas/Providencia*, indole-positive *Proteus/ Acinetobacter/Morganella*, *Citrobacter*, *Enterobacter* or *Hafnia*) [20, 21]. In total, we found no significant increase in gram-negative peritonitis over the three decades. However, third generation cephalosporin resistant gram-negative rods (3GCR-GN) increased in the last period from 2004–2014. ESBL-producing *E*. *coli* peritonitis is associated with a worse outcome [22] and must therefore be considered in daily clinical practice. Both first line regimens for gram-negative organisms have had similar susceptibility profiles for gram-negative organisms. Susceptibility to ceftazidim varied between 100% and 84%. Sensitivity to gentamicine varied between 82% and 94%. This indicates the need for alternative antibiotic regimens if first line therapy fails or *in vitro* testing indicates a resistance to one of the antibiotics. The current rise in 3GCR-GN indicated the need of a carbapenem antibiotic regimen in the treatment of PD-associated peritonitis. A small prospective open-label study showed that monotherapy with imipenem/cilastatin has similar efficacy compared to cefazolin plus ceftazidime or netilmycin in the treatment of PD-associated peritonitis [23]. To limit the risk of under- and overdosing, routine measurement of blood concentrations should be performed [24]. However, randomized controlled trials for the use of carbapenems in PD peritonitis are lacking. The ISPD outlines the importance of a surveillance program in each PD center to adapt the empiric therapy to the local resistance spectrum [7]. As a consequence of our local antimicrobial surveillance we changed the empiric therapy in PD patients with peritonitis from “cefazolin i.p. and gentamycin i.p.” to “vancomycin i.p. and gentamycin i.p. in 2014.

Hence, our data illustrate the appropriateness of ISPD guidelines recommending center-specific selection of empirical therapy. Furthermore, it must be noted that *in vitro* sensitivity testing is often not sufficient in clinical practice.

Our study has several limitations. First we presented data from a single center in southern Germany with an in-hospital patient cohort. The total number of episodes was different in the three decades due to an increase number in patients treated by our center. Second, different microbiological techniques for etiologic identification of organisms and susceptibility testing were used during the three decades. Third all *in vitro* susceptibility was defined according to CLSI but limit specifications of the CLSI varied over time. Fourthly the available data does not allow a differentiation between ESBL and AmpC beta-lactamases ESBL producing strains could only suspect by resistance to third generation cephalosporins.

Fifths, due to the long study period and the conception of our center as a referral center, the clinical data regarding peritonitis rate and PD details are incomplete. Very recently, Esch et al. reported the incidence of peritonitis, causative pathogens and clinical outcomes from 1979 to July 2010 from a PD center in the Netherlands including PD details, but without susceptibility rates [25].

In conclusion, we found a decrease in *S*. *aureus* and CNS associated peritonitis over the three decades, whereas the proportion of MRSE increased during the period. MRSA peritonitis is a rare event and peritonitis caused by 3GCR-GN increased. Susceptibility to cefazolin in gram-positive organisms decreased over the three decades but vancomycin was effective in all episodes except in VRE. Susceptibility to gentamicin and ceftazidim was comparable. Our data suggests that it is necessary to implement local antibiotic stewardship strategies in PD programs.
